# Assessing the Lifetime Performance Index with Digital Inferences of Power Hazard Function Distribution Using Progressive Type-II Censoring Scheme

**DOI:** 10.1155/2022/6467724

**Published:** 2022-02-16

**Authors:** Rashad M. EL-Sagheer, Taghreed M. Jawa, Neveen Sayed-Ahmed

**Affiliations:** ^1^Department of Mathematics, Faculty of Science, A1-Azhar University, Nasr City 11 884, Cairo, Egypt; ^2^Department of Mathematics, College of Science, Taif University, P.O. Box 11 099, Taif 21 944, Saudi Arabia

## Abstract

This paper deals with estimating the lifetime performance index. The maximum likelihood (ML) and Bayesian estimators for lifetime performance index *C*_*L*_*X*__ where *L*_*X*_ is the lower specification limit are derived based on progressive type-II censored (Prog-Type-II-C) sample from two-parameter power hazard function distribution (PHFD). Knowing the lower specification limit, the MLE of *C*_*L*_*X*__ is applied to construct a new hypothesis testing procedure. Bayesian estimator of *C*_*L*_*X*__ is also utilized to develop a credible interval. Also, the relationship between the *C*_*L*_*X*__ and the conforming rate of products is investigated. Moreover, the Bayesian test to evaluate the lifetime performance of units is proposed. A simulation study and illustrative example based on a real dataset are discussed to evaluate the performance of the two tests.

## 1. Introduction

Process capability analysis plays an important role in the quality control field to measure the performance of process in the industry. The lifetime performance index ( process capability index or (PCI)) has been proposed by Montgomery [1] and Kane [2]. The index is used to assess the lifetime performance of electronic units. All process capability indices (PCIs) have been discussed using the assumption that the lifetime of products (units) follows a normal distribution. Nevertheless, the assumption of normality for many processes in industry and business cannot be valid. A lot of papers were studied on the statistical inference for *C*_*L*_*X*__ based on various types of censored and progressive censored data for different models of which [3–17] dealt with progressive censoring from various points of view considering several lifetime distributions and its applications. Soliman et al. [18, 19] discussed assessing the life time performance index for exponentiated Frechet distribution using Prog-Type-II-C and progressive first failure censoring scheme. Recently, Wu et al. [20] introduced the reliability sampling design for *C*_*L*_*X*__ of Gompertz lifetime distribution under progressive type-I interval censoring, Zhang and Gui [21] studied the statistical inference for *C*_*L*_*X*__ with Pareto distribution on the basis of general progressive type-II censored sample, Wu et al. [22] proposed experimental design for *C*_*L*_*X*__ of Weibull products based on the progressive type-I interval censored sample, and Hassan and Assar [23] discussed assessing *C*_*L*_*X*__ of Burr type-III distribution under progressive type-II censoring.

The Prog-Type-II-C scheme is of use in this paper [11–17], which can be characterized as follows: we presume that *n* units are placed on a life testing experiment. Let *X*_*i*_, *i*=1,2,…, *n* be continuous identically distributed failure times of these units. The following scheme (*R*_1_, *R*_2_,…, *R*_*m*_) are considered where *m* < *n* and *R*_1_, *R*_2_,…, *R*_*m*_ all are previously fixed and ∑_*i*=1_^*m*^*R*_*i*_+*m*=*n*. At the first failure time *X*_1_, *R*_1_ units can be removed randomly from the remaining *n* − 1 surviving units. Then, immediately at the second failure time *X*_2_, *R*_2_ surviving units are randomly removed. This technique continues until the time of *m*^*th*^ failure *X*_*m*_. Then, the remaining units *R*_*m*_ are deleted.

Let *X*_1:*m*:*n*_^(*R*_1_,…,*R*_*m*_)^, *X*_2:*m*:*n*_^(*R*_1_,…,*R*_*m*_)^,…, *X*_*m*:*m*:*n*_^(*R*_1_,…,*R*_*m*_)^ be the prog-Type-II-C sample with size *m* from a sample of size *n*. One can easily note the following:

- If *R*_1_=*R*_2_=⋯=*R*_*m*−1_=0, then type-II right censored order statistics are deduced
- If *R*_1_=*R*_2_=⋯=*R*_*m*_=0, then ordinary order statistics are obtained

In this paper, the lifetimes of units have the two-parameter PHFD(*α*, *β*). Statistical inference for the unknown *C*_*L*_*X*__ is developed based on Prog-Type-II-C data. Mugdadi [24] proposed the two-parameter PHFD(*α*, *β*). The hazard rate function (hrf), cumulative distribution function (cdf), probability density function (pdf), and survival function (sf) are given respectively by

(1) $$\begin{array}{c} h\left(x\right)=\alpha x^{\beta },\;\alpha >0,\;\beta >-1,\;x>0, \end{array}$$

(2) $$\begin{array}{c} F_{X}\left(x;\alpha ,\beta \right)=1-\exp\left\{-\frac{\alpha }{\beta +1}x^{\beta +1}\right\},\;\alpha >0,\;\beta >-1,\;x>0, \end{array}$$

(3) $$\begin{array}{c} f_{X}\left(x;\alpha ,\beta \right)=\alpha x^{\beta }\exp\left\{-\frac{\alpha }{\beta +1}x^{\beta +1}\right\},\;\alpha >0,\;\beta >-1,\;x>0, \end{array}$$

(4) $$\begin{array}{c} S_{X}\left(x\right)=\exp\left\{-\frac{\alpha }{\beta +1}x^{\beta +1}\right\},\;\alpha >0,\;\beta >-1,\;x>0, \end{array}$$

where *α* and *β* are the scale and shape parameters, respectively. It is clear that

- For *β* > 0, the PHFD has an increasing hrf
- For −1 < *β* < 0, it has decreasing hrf

Weibull, Rayleigh, and exponential distributions can be deduced as special cases of the PHFD as follows:

1. If *β*=*α* − 1, then PHFD reduces to Weibull (*α*, 1)
2. If *α*=1/*λ*^2^ and *β*=1, then PHFD reduces to Rayleigh (*λ*)
3. If *β*=0, then PHFD reduces to exponential distribution with mean 1/*α*

Therefore, the results in this paper are valid for Weibull, Rayleigh, and exponential distributions. The PHFD(*α*, *β*) has the following properties:

(1) The expected value of *X* is
  (5) $$\begin{array}{c} E\left(X\right)=\int_{0}^{\infty }xf_{X}\left(x\right)\text{d}x\\ =\alpha \int_{0}^{\infty }x^{\beta +1}\exp\left\{-\frac{\alpha }{\beta +1}x^{\beta +1}\right\}\text{d}x\\ ={\left(\frac{\alpha }{\beta +1}\right)}^{-1/\beta +1}\Gamma \left(\frac{\beta +2}{\beta +1}\right), \end{array}$$
where Γ(.) is the complete gamma function.
(2) The expected value of *X*^2^ is
  (6) $$\begin{array}{c} E\left(X^{2}\right)=\int_{0}^{\infty }x^{2}f_{X}\left(x\right)\text{d}x\\ =\alpha \int_{0}^{\infty }x^{\beta +2}\exp\left\{-\frac{\alpha }{\beta +1}x^{\beta +1}\right\}\text{d}x\\ ={\left(\frac{\alpha }{\beta +1}\right)}^{-2/\beta +1}\Gamma \left(\frac{\beta +3}{\beta +1}\right). \end{array}$$
(3) The standard deviation

(7) $$\begin{array}{c} \sigma =\sqrt{E\left(X^{2}\right)-{\left[E\left(X\right)\right]}^{2}}\\ =\gamma_{2}{\left(\frac{\alpha }{\beta +1}\right)}^{-1/\beta +1}, \end{array}$$

where

(8) $$\begin{array}{c} \gamma_{2}=\sqrt{\Gamma \left(\frac{\beta +3}{\beta +1}\right)-{\left(\gamma_{1}\right)}^{2}},\;\gamma_{1}=\Gamma \left(\frac{\beta +2}{\beta +1}\right). \end{array}$$

The main aim of this article is to secure the ML and Bayesian estimators for *C*_*L*_*X*__ in view of Prog-Type-II-C sample from PHFD(*α*, *β*) where *β* is known parameter. The ML estimator of *C*_*L*_*X*__ is then used to construct a new hypothesis testing based on known *L*_*X*_. The Bayesian test is also proposed to evaluate *C*_*L*_*X*__ of units.

The rest of this paper is organized as follows: Section 2 contains a derivation of *C*_*L*_*X*__.  Section 3 discusses the relationship between *C*_*L*_*X*__ and the conforming rate *P*_*r*_ of products. The ML estimate of *C*_*L*_*X*__ and some of the corresponding statistical properties are investigated in Section 4. Bayesian approach of *C*_*L*_*X*__ in the presence of gamma prior distribution is presented in Section 5, and Section 6 improves testing procedures for *C*_*L*_*X*__. A real dataset has been analyzed to illustrate the use of the testing procedure based on the proposed estimators in Section 7. Simulation studies are given in Section 8. Finally, conclusion appears in Section 9.

## 2. The Lifetime Performance Index *C*_*L*_*X*__

Let *X* be the lifetime of products which has PHFD(*α*, *β*) with pdf and cdf given in (2) and (3), respectively. It is known to the consumer that the high-quality product is the one that lasts for a longer lifetime, and therefore, the lifetime is the characteristic of the high-quality product. Moreover, in order for the product to be profitable from a financial point of view and satisfactory to customers as well, the lifetime required to exceed *L*_*X*_ unit times. Montgomery [1] suggested a capability index *C*_*L*_*X*__ to measure the features of a product that has better and higher quality. Then, *C*_*L*_*X*__ is defined as

(9) $$\begin{array}{c} C_{L_{X}}=\frac{\mu -L_{X}}{\sigma }, \end{array}$$

where *μ*=*E*(*X*) denotes the mean lifetime, $\sigma =\sqrt{E\left(X^{2}\right)-{\left[E\left(X\right)\right]}^{2}}$ represents the lifetime standard deviation, and *L*_*X*_ indicates the lower specification limit. Under the condition that *X* has PHFD(*α*, *β*), then from (5), (7), and (9), the lifetime performance index *C*_*L*_*X*__ is written as

(10) $$\begin{array}{c} C_{L_{X}}=\frac{1}{\gamma_{2}}\left[\gamma_{1}-{\left(\frac{\alpha }{\beta +1}\right)}^{1/\beta +1}L_{X}\right], \end{array}$$

where *γ*_1_ and *γ*_2_ are given in (8).

## 3. Conforming Rate

If the new lifetime of a product (or item) *X* exceeds the lower specification limit *L*_*X*_ (i.e., *X* > *L*_*X*_), then the product is labelled as a conforming productA. Otherwise, the product is labelled as a nonconforming product. Therefore, the ratio of the conforming product is known as the conforming probability, or sometimes also called conforming rate *P*_*r*_, and can be defined as

(11) $$\begin{array}{c} P_{r}=P\left(X\geq L_{X}\right)\\ =\exp\left\{-\frac{\alpha }{\beta +1}{\left[{\left(\frac{\alpha }{\beta +1}\right)}^{\left(-1/\beta +1\right)}\left(\gamma_{1}-\gamma_{2}C_{L_{X}}\right)\right]}^{\beta +1}\right\}. \end{array}$$

Obviously, a strictly positive relationship exists between *P*_*r*_ and *C*_*L*_*X*__, for given *β* > −1. Thus, the higher the index value *C*_*L*_*X*__ gives the higher of the conforming rate *P*_*r*_.  Table 1 lists some numerical values of *C*_*L*_*X*__ and the corresponding *P*_*r*_ for given *β*=0.332 and *α*=1.1835, which can be obtained by using the graphical method, the *P*_*r*_ can be calculated by (11) for given *α*, *β*, and *C*_*L*_*X*__.

## 4. Maximum Likelihood Estimator of *C*_*L*_*x*__

Let *X*_1:*m*::*n*_ < *X*_2:*m*::*n*_ < ⋯<*X*_*m*:*m*::*n*_ be a Prog-Type-II-C sample from PHFD(*α*, *β*), with pdf and cdf as defined in equations (2) and (3), respectively. We denote the observed values of such Prog-Type-II-C sample by *x*_*i*_ , *i* =1,2,…, *m*. According to Balakrishnan and Sandhu [12], the likelihood function of Prog-Type-II-C with scheme *R*_*i*_ ≥ 0, *i*=1,2,…, *m* is

(12) $$\begin{array}{c} L\left(\alpha ,\beta ;\underline{x}\right)=C\prod_{i=1}^{m}f_{X}\left(x;\alpha ,\beta \right){\left[1-F_{X}\left(x;\alpha ,\beta \right)\right]}^{R_{i}}, \end{array}$$

where

(13) $$\begin{array}{c} C=n\left(n-R_{1}-1\right)\left(n-R_{1}-R_{2}-2\right)\ldots \left(n-\sum_{i=1}^{m-1}R_{i}-m+1\right). \end{array}$$

Substituting from (2) and (3) into (12), the likelihood function for $\underline{x}$ is given by

(14) $$\begin{array}{c} L\left(\alpha ,\beta ;\underline{x}\right)=C\alpha^{m}\left[\prod_{i=1}^{m}x_{i}^{\beta }\right]\exp\left\{-\frac{\alpha }{\beta +1}\sum_{i=1}^{m}\left(R_{i}+1\right)x_{i}^{\beta +1}\right\}. \end{array}$$

The log-likelihood function can be obtained from (14) as

(15) $$\begin{array}{c} \ell \left(\alpha ,\beta ;\underline{x}\right)=\log\;C+m\;\log\left(\alpha \right)+\beta \sum_{i=1}^{m}\log\left(x_{i}\right)-\frac{\alpha }{\beta +1}\sum_{i=1}^{m}\left(R_{i}+1\right)x_{i}^{\beta +1}. \end{array}$$

Consequently, for known *β*, the likelihood equation of *α* is obtained as

(16) $$\begin{array}{c} \frac{\partial \ell \left(\alpha ,\beta ;\underline{x}\right)}{\partial \alpha }=\frac{m}{\alpha }-\frac{1}{\beta +1}\sum_{i=1}^{m}\left(R_{i}+1\right)x_{i}^{\beta +1}=0. \end{array}$$

Therefore, the ML estimator of *α* is

(17) $$\begin{array}{c} {\hat{\alpha }}_{\text{ML}}=m{\left[\frac{1}{\beta +1}\sum_{i=1}^{m}\left(R_{i}+1\right)x_{i}^{\beta +1}\right]}^{-1}. \end{array}$$

Thus, according to Zehna [25], the MLE of *C*_*L*_*X*__ becomes

(18) $$\begin{array}{c} {\hat{C}}_{L_{X}}^{\text{ML}}=\frac{1}{\gamma_{2}}\left[\gamma_{1}-{\left(\frac{m}{W}\right)}^{1/\beta +1}L_{X}\right], \end{array}$$

where *W*=∑_*i*=1_^*m*^(*R*_*i*_+1)*x*_*i*_^*β*+1^ and *γ*_1_ and *γ*_2_ are given in (8).

## 5. Bayes Estimation of *C*_*L*_*X*__

In this section, based on Prog-Type-II-C sample under PHFD(*α*, *β*) with known *β*, the Bayesian method for deriving estimates of both *α* and *C*_*L*_*X*__ is discussed. In lifetime data analysis, such prior knowledge is usually summarized into a prior density, denoted by *π*(*α|a*, *b*). We consider the conjugate prior distribution to be gamma distribution with the pdf as

(19) $$\begin{array}{c} \pi \left(\alpha |a,b\right)=\frac{b^{a}}{\Gamma \left(a\right)}\alpha^{a-1}\exp\left\{-b\alpha \right\},\;a>0,\;b>0,\;\alpha >0, \end{array}$$

where the hyperparameters *a* and *b* are chosen to reflect prior knowledge about the unknown parameter *α*. Based on (14) and (19), we can obtain the posterior density function of *α*, given the data as

(20) $$\begin{array}{c} \pi^{*}\left(\alpha |\underline{x}\right)=\frac{W^{\prime m+a}}{\Gamma \left(m+a\right)}\alpha^{m+a-1}\exp\left\{-\alpha W{}^{\prime }\right\}, \end{array}$$

for *α* > 0 and zero elsewhere, where

(21) $$\begin{array}{c} W{}^{\prime }=b+\frac{1}{\beta +1}\sum_{i=1}^{m}\left(R_{i}+1\right)x_{i}^{\beta +1}. \end{array}$$

Based on a SELF $\ell \left(\alpha ,\hat{\alpha }\right)={\left(\hat{\alpha }-\alpha \right)}^{2}$ and (20), the Bayesian estimation for the parameter *α* becomes

(22) $$\begin{array}{c} {\hat{\alpha }}_{BS}=E\left[\alpha |\underline{x}\right]\\ =\frac{m+a}{W{}^{\prime }}. \end{array}$$

Hence, the Bayes estimator ${\hat{C}}_{L_{X}}^{BS}$ of *C*_*L*_*X*__ can be written as

(23) $$\begin{array}{c} {\hat{C}}_{L_{X}}^{BS}=E\left[C_{L_{X}}|\underline{x}\right]\\ =\frac{1}{\gamma_{2}}\left[\gamma_{1}-\frac{\Gamma \left(m+a+1/\beta +1\right)}{\Gamma \left(m+a\right)}{\left(\frac{1}{\beta +1}\right)}^{1/\beta +1}L_{X}W^{\prime^{-1/\beta +1}}\right], \end{array}$$

where *W*′ is given in (21).

**Lemma 1 .**

If *W*′=*b*+1/*β*+1∑_*i*=1_^*m*^(*R*_*i*_+1)*x*_*i*_^*β*+1^, then 2*αW*′ follows the chi-square distribution with 2(*m*+*a*) degrees of freedom, denoted by 2*αW*′∼*χ*_2(*m*+*a*)_^2^.

**Proof**

Let *α*=*y*/2*W*′, then ‖*J*_*y*_‖=1/2*W*′ (see Casella and Berger [26], pp.); we obtain the density function of *y* as

(24) $$\begin{array}{c} f_{Y}\left(y\right)=\pi^{*}\left(\frac{y}{2W\prime }|\underline{x}\right)\left\|J_{y}\right\|\\ =\left[\frac{W^{\prime m+a}}{\Gamma \left(m+a\right)}{\left(\frac{y}{2W{}^{\prime }}\right)}^{m+a-1}\left(\exp\left\{-\frac{y}{2}\right\}\right)\right]\left(\frac{1}{2W{}^{\prime }}\right)\\ =\frac{1}{2^{\left(2\left(m+a\right)/2\right)\;}\Gamma \left(2\left(m+a\right)/2\right)}y^{\left(2\left(m+a\right)/2\right)-1}\left(\exp\left\{-\frac{y}{2}\right\}\right)\\ =\frac{1}{2^{\left(m+a\right)}\Gamma \left(m+a\right)}y^{m+a-1}\left(\exp\left\{-\frac{y}{2}\right\}\right). \end{array}$$

Therefore, *Y*=2*αW*′ ~ *χ*_2(*m*+*a*)_^2^.

## 6. Testing Procedure for *C*_*L*_*X*__

This section is devoted to construct a statistical testing procedure to evaluate whether *C*_*L*_*X*__ reaches the required level. Credible and confidence intervals for *C*_*L*_*X*__ are calculated to objectively evaluate whether *C*_*L*_*X*__ adheres to the required level. The null and the alternative hypotheses *H*_0_ (the product is unreliable) and *H*_1_ (the product is reliable) respectively can be written as

(25) $$\begin{array}{c} H_{0}:C_{L_{X}}\leq c,\;H_{1}:C_{L_{X}}>c, \end{array}$$

where *c* denotes the lower bound of *C*_*L*_*X*__.

In the Bayesian approach, for given specified significance level *δ*, a 100(1 − *δ*)% one-sided credible interval (CRI) for *C*_*L*_*X*__ is derived as follows. Since *β* is known, by using the pivotal quantity 2*αW*′ ~ *χ*_2(*m*+*a*)_^2^, and the lower (1 − *δ*) percentile of *χ*_2(*m*+*a*)_^2^ denoted by *χ*_(1 − *δ*, 2(*m*+*a*))_^2^, we have

(26) $$\begin{array}{c} \left.\begin{array}{c} P\left(2\alpha W{}^{\prime }\leq \chi_{\left(1-\delta ,2\left(m+a\right)\right)}^{2}\right)=1-\delta ,\\ \Rightarrow P\left(\frac{1}{\gamma_{2}}\left[\gamma_{1}-{\left(\frac{\alpha }{\beta +1}\right)}^{1/\beta +1}L_{X}\right]\geq \frac{1}{\gamma_{2}}\left[\gamma_{1}-{\left(\frac{\chi_{\left(1-\delta ,2\left(m+a\right)\right)}^{2}}{2W\prime \left(\beta +1\right)}\right)}^{1/\beta +1}L_{X}\right]\right)=1-\delta ,\\ \Rightarrow P\left(C_{L_{X}}\geq \frac{1}{\gamma_{2}}\left[\gamma_{1}-{\left(\frac{\chi_{\left(1-\delta ,2\left(m+a\right)\right)}^{2}}{2W\prime \left(\beta +1\right)}\right)}^{1/\beta +1}L_{X}\right]\right)=1-\delta ,\\ \Rightarrow P\left(C_{L_{X}}\geq \frac{1}{\gamma_{2}}\left[\gamma_{1}-{\left(\frac{\chi_{\left(1-\delta ,2\left(m+a\right)\right)}^{2}}{2\left(m+a\right)\left(\beta +1\right)}\right)}^{1/\beta +1}\left(\gamma_{1}-\gamma_{2}{\hat{C}}_{L_{X}}^{BS}\right)\right]\right)=1-\delta , \end{array}\right\} \end{array}$$

Here, *γ*_1_, *γ*_2_, *W*′, and ${\hat{C}}_{L_{X}}^{BS}$ are given by (8), (21), and (23), respectively. Therefore, the level 100(1 − *δ*)% lower credible bound for *C*_*L*_*X*__ can be written as

(27) $$\begin{array}{c} {\underline{LB}}_{BS}=\frac{1}{\gamma_{2}}\left[\gamma_{1}-{\left(\frac{\chi_{\left(1-\delta ,2\left(m+a\right)\right)}^{2}}{2\left(m+a\right)\left(\beta +1\right)}\right)}^{1/\beta +1}\left(\gamma_{1}-\gamma_{2}{\hat{C}}_{L_{X}}^{BS}\right)\right]. \end{array}$$

At the same time, we derive the maximum likelihood approach by using 2*αW* ~ *χ*_(2*m*)_^2^, where *W*=∑_*i*=1_^*m*^(*R*_*i*_+1)*x*_*i*_^*β*+1^. The 100(1 − *δ*)% one-sided confidence interval (CI) for *C*_*L*_*X*__ is then given by

(28) $$\begin{array}{c} C_{L_{X}}\geq \frac{1}{\gamma_{2}}\left[\gamma_{1}-{\left(\frac{\chi_{\left(1-\delta ,2m\right)}^{2}}{2m\left(\beta +1\right)}\right)}^{1/\beta +1}\left(\gamma_{1}-\gamma_{2}{\hat{C}}_{L_{X}}^{ML}\right)\right], \end{array}$$

where ${\hat{C}}_{L_{X}}^{ML}$ is given in (18). Hence, the level 100(1 − *δ*)% lower confidence bound for *C*_*L*_*X*__ can be derived as

(29) $$\begin{array}{c} {\underline{LB}}_{ML}=\frac{1}{\gamma_{2}}\left[\gamma_{1}-{\left(\frac{\chi_{\left(1-\delta ,2m\right)}^{2}}{2m\left(\beta +1\right)}\right)}^{1/\beta +1}\left(\gamma_{1}-\gamma_{2}{\hat{C}}_{L_{X}}^{ML}\right)\right]. \end{array}$$

The proposed testing procedure about *C*_*L*_*X*__ in the Bayesian approach can be organized as follows.

**Step 1 .**

Specify the lower lifetime limit *L*_*X*_ for products and performance index value *c*; then, the testing null hypothesis *H*_0_: *C*_*L*_*X*__ ≤ *c* and the alternative hypothesis *H*_1_: *C*_*L*_*X*__ > *c* are created.

**Step 2 .**

Determine a significance level *δ*

**Step 3 .**

Compute the level 100(1 − *δ*)% one-sided CRI $\left[{\underline{LB}}_{BS},\infty \right)$ for *C*_*L*_*X*__

**Step 4 .**

The decision rule of the statistical test is provided as follows: if the performance index value $c\notin \left[{\underline{LB}}_{BS},\infty \right)$, it is concluded that the lifetime performance index of the product meets the required level

The ML approach uses 100(1 − *δ*)% one-sided CI $\left[{\underline{LB}}_{ML},\infty \right)$ for *C*_*L*_*X*__ instead of 100(1 − *δ*)% one-sided CRI $\left[{\underline{LB}}_{BS},\infty \right)$ to see if the product performance meets the required level.

## 7. Application to Real-Life Data

**Figure 1:**
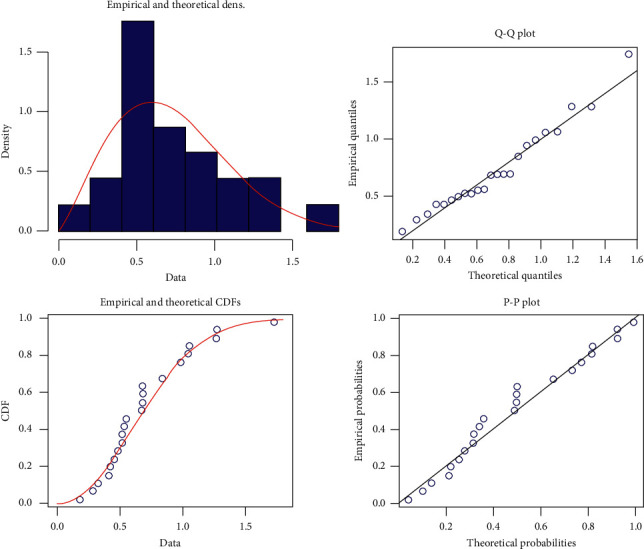
Empirical, Q-Q, and P–P plots of PHFD for the real dataset.

The combination between theoretical and application methods plays an important role in modern statistical problems. In this section, a theoretical technique is applied to a set of real data for illustration of the proposed procedures. We consider a real dataset given by Leiblein and Zelen [27], which was used recently by Cho et al. [28]. For the purpose of the goodness-of-fit test, the Kolmogorov–Smirnov distance between the empirical and the fitted distribution functions has been computed. It is 0.150 81, and the associated *p*-value is 0.672 3. Hence, the *p*-value for Kolmogorov–Smirnov test has the highest value for the dataset. This leads us to conclude that PHFD is the best fit for the real dataset. Empirical, *Q* − *Q*, and *P* − *P* plots are shown in Figure 1, which clear that the PHFD fits the data very well. This set of real data represents 23 observed failure times. The Prog-Type-II-C scheme was conducted with *n*=23, *m*=18, and the censoring scheme *R*_*i*_, *i*=1,…, 18 is generated from the original data. The observed data and removed numbers are reported in Table 2, where $\hat{\alpha }=1.1835$ and $\hat{\beta }=0.332$, which can be obtained by using the graphical method introduced by Balakrishnan and Kateri [29].

In the Bayesian approach, we assumed that the values of the hyperparameters *a*=0.001 and *b*=0.001. Under the Prog-Type–II–C sample, the proposed testing procedure for *C*_*L*_*X*__ can be performed in the following steps:

(i) Step 1 : *L*_*X*_ is assumed to be 0.441 1. To meet the product purchasers' concerns regarding operational performance, the *P*_*r*_ of products is required to exceed 82%. Referring to Table 1, the *C*_*L*_*X*__ value is required to exceed 0.90. Thus, *c*=0.90 and *H*_0_: *C*_*L*_*X*__ ≤ 0.90 vs. *H*_1_ : *C*_*L*_*X*__ > 0.90 is constructed.
  Step 2 : let the significance level be given by *δ*=0.05.
(ii) Step 3 : the lower bound ${\underline{LB}}_{BS}$ of the 95% one-sided CRI for *C*_*L*_*X*__ is
  (30) $$\begin{array}{c} {\underline{LB}}_{BS}=\frac{1}{0.6970}\left[0.9192-{\left(\frac{\chi_{\left(1-0.05,2\left(18+1\right)\right)}^{2}}{2\left(18+1\right)\left(0.332+1\right)}\right)}^{1/0.332+1}\left[0.9192-\left(0.6970\right)\left(0.9427\right)\right]\right]\\ =0.9274, \end{array}$$
where *γ*_1_=0.9192 and *γ*_2_=0.6970 are calculated according to (8).
  Step 4 : because *c*=0.90 ∉ [0.9274, *∞*), the null hypothesis *H*_0_: *C*_*L*_*X*__ ≤ 0.90 is rejected.

In the ML approach, the lower bound ${\underline{LB}}_{\text{ML}}$ of the 95% one-sided CI for *C*_*L*_*X*__ is

(31) $$\begin{array}{c} {\underline{LB}}_{\text{ML}}=\frac{1}{0.6970}\left[0.9192-{\left(\frac{\chi_{\left(1-0.05,\left(2\times 18\right)\right)}^{2}}{\left(2\times 18\right)\left(0.332+1\right)}\right)}^{1/0.332+1}\left[0.9192-\left(0.6970\right)\left(0.9382\right)\right]\right]\\ =0.9201. \end{array}$$

Because of the performance index value *c*=0.90 ∉ [0.9201, *∞*), we reject *H*_0_: *C*_*L*_*X*__ ≤ 0.90. To sum up, *C*_*L*_*X*__ of products meets the required quality level for the Bayesian and ML approaches.

## 8. Monte Carlo Simulation Study

To compare the ML and Bayes estimators for *C*_*L*_*X*__ which are proposed in previous sections, Monte Carlo simulations were performed utilizing 1000 Prog-Type-II-C samples for each simulation. All obtained simulation study results were performed using Mathematica Ver. 13. The mean square error (MSE) is used to compare the estimators. The samples were generated from PHFD using (*α*, *β*)=(1.092, 0.332) , with different *n*, *m*, and hyperparameters (*a*, *b*). In this study, we used different censoring schemes as follows.

**Scheme 1 .**

*R*_1_=*n* − *m*, *R*_*i*_=0 for *i* ≠ 1.

**Scheme 2 .**

*R*_*m*+1/2_=*n* − *m*, *R*_*i*_=0 for *i* ≠ *m*+1/2, if *m* is odd, and *R*_*m*/2_=*n* − *m*, *R*_*i*_=0 for *i* ≠ *m*/2, if *m* is even.

**Scheme 3 .**

*R*_*m*_=*n* − *m*, *R*_*i*_=0 for *i* ≠ *m*.

Based on the lower lifetime limit *L*_*X*_=0.022, the results of MSEs of the ML, Bayes estimates, and coverage probabilities (CPs) of the 95% CRI and CI for *C*_*L*_*X*__ are presented in Table 3.

## 9. Conclusions

This paper aims to construct Bayesian and non-Bayesian approaches, with various estimators for *C*_*L*_*X*__ with the Prog-Type-II-C sample from PHFD(*α*, *β*). Under the condition of known *L*_*X*_, the MLEs and Bayesian estimator of *C*_*L*_*X*__ are then used to develop the new hypotheses testing procedure. From the application of real-life data, it turns out that the suggested test can be carried out easily so that we can assess whether the product quality (lifetime of products) meets the requirements of customers and at the same time brings abundant profit. Furthermore, it indicates that these assessing methods are practical and feasible. An elaborate simulation study was conducted for different sample sizes *n*, *m* and different censoring schemes (*I*, *II*, *III*) to evaluate the performance of these proposed procedures. For the point estimations, MSE was compared. For the interval estimations, the coverage rates were obtained. According to the tabulated results of the estimates in Table 3, the following concluding remarks can be drawn:

1. For the censoring scheme *I*, the MSE values of all estimates decrease as *m*/*n* increases which is consistent with the statistical theory that the larger the sample size, the more accurate of the estimate
2. Generally, the performance of the Bayes estimators is better than MLE for the all considered cases because it has the smallest MSE
3. The CPs of both ACIs and CRI are sensibly satisfactory and in most cases are near to the nominal confidence level of 95%

## Figures and Tables

**Table 1:** The numerical values of *C*_*L*_*X*__ vs. the corresponding *P*_*r*_.

*C*_*L*_*X*__	*P*_*r*_
−*∞*	0.000 000
−5.00	0.000 743
−4.50	0.001 572
−4.00	0.003 256
−3.50	0.006 594
−3.00	0.013 039
−2.50	0.025 127
−2.00	0.047 086
−1.50	0.085 566
−1.00	0.150 250
−0.50	0.253 703
0.00	0.409 062
0.05	0.427 827
0.10	0.447 191
0.15	0.467 153
0.20	0.487 704
0.25	0.508 837
0.30	0.530 539
0.35	0.552 793
0.40	0.575 579
0.45	0.598 870
0.50	0.622 636
0.55	0.646 840
0.60	0.671 437
0.65	0.696 375
0.70	0.721 594
0.75	0.747 022
0.80	0.772 575
0.85	0.798 156
0.90	0.823 649
0.95	0.848 916
1.00	0.873 793
1.05	0.898 078
1.10	0.921 517
1.15	0.943 778
1.20	0.964 394
1.25	0.982 622
1.30	0.996 872
1.31	0.998 850

**Table 2:** Prog-Type-II-C sample from Leiblein and Zelen [19].

*i*	1	2	3	4	5	6	7	8	9
*x*_*i*,18,23_	0.178 8	0.330 0	0.415 2	0.456 0	0.484 8	0.518 6	0.519 6	0.541 2	0.555 6
*R*_*i*_	0	0	0	0	0	0	0	0	0
*i*	10	11	12	13	14	15	16	17	18
*x*_*i*,18,23_	0.678 0	0.686 4	0.841 2	0.931 2	0.986 4	1.058 4	1.279 2	1.280 4	1.734 0
*R*_*i*_	0	0	1	0	1	0	1	0	2

**Table 3:** MSEs and CPs of the MLEs and Bayes estimates for *C*_*L*_*X*__.

N	m	Sc.	ML	Bayes	Bayes	ML	Bayes	Bayes
			Mean squared errors (MSEs)			Coverage probabilities (CPs)		
				*a* = 1, *b* = 2	*a* = 2, *b* = 3		*a* = 1, *b* = 2	*a* = 2, *b* = 3
25	15	I	0.000 088 9	0.000 080 4	0.000 072 9	0.947	0.949	0.956
		II	0.000 091 9	0.000 084 9	0.000 075 0	0.939	0.948	0.953
		III	0.000 097 2	0.000 089 7	0.000 081 1	0.945	0.939	0.948

30	20	I	0.000 083 7	0.000 077 4	0.000 068 7	0.941	0.943	0.939
		II	0.000 086 6	0.000 079 4	0.000 071 3	0.952	0.948	0.947
		III	0.000 092 2	0.000 084 2	0.000 079 2	0.954	0.953	0.955

30	25	I	0.000 076 7	0.000 069 9	0.000 061 8	0.962	0.954	0.963
		II	0.000 082 3	0.000 074 2	0.000 066 6	0.941	0.951	0.947
		III	0.000 089 1	0.000 078 5	0.000 072 9	0.939	0.940	0.951

50	30	I	0.000 071 6	0.000 062 6	0.000 059 3	0.944	0.959	0.945
		II	0.000 075 7	0.000 068 4	0.000 063 1	0.940	0.936	0.942
		III	0.000 081 4	0.000 073 2	0.000 067 5	0.945	0.941	0.939

50	40	I	0.000 067 3	0.000 058 4	0.000 055 8	0.960	0.952	0.962
		II	0.000 071 5	0.000 062 6	0.000 058 5	0.954	0.958	0.957
		III	0.000 076 8	0.000 066 9	0.000 062 8	0.938	0.940	0.948

70	50	I	0.000 059 9	0.000 049 8	0.000 046 7	0.951	0.949	0.947
		II	0.000 063 3	0.000 054 3	0.000 051 6	0.944	0.945	0.952
		III	0.000 067 7	0.000 058 5	0.000 055 9	0.938	0.941	0.949

90	60	I	0.000 038 4	0.000 029 9	0.000 025 8	0.955	0.961	0.954
		II	0.000 043 6	0.000 036 3	0.000 033 4	0.948	0.939	0.945
		III	0.000 047 1	0.000 038 2	0.000 035 1	0.954	0.940	0.940

90	70	I	0.000 022 8	0.000 019 6	0.000 014 9	0.956	0.953	0.958
		II	0.000 028 7	0.000 023 4	0.000 018 5	0.945	0.943	0.951
		III	0.000 034 1	0.000 028 5	0.000 023 2	0.942	0.941	0.939

## Data Availability

The data used are theoretically generated from the laws used in the manuscript.
